# The Group Right to Mutual Privacy

**DOI:** 10.1007/s44206-023-00051-5

**Published:** 2023-06-06

**Authors:** Anuj Puri

**Affiliations:** Tilburg Institute for Law, Technology, and Society (TILT), Tilburg Law School, Tilburg, the Netherlands

**Keywords:** Mutual privacy, Group right to mutual privacy, Privacy as a public good

## Abstract

Contemporary privacy challenges go beyond individual interests and result in collective harms. To address these challenges, this article argues for a collective interest in Mutual Privacy which is based on our shared genetic, social, and democratic interests as well as our common vulnerabilities against algorithmic grouping. On the basis of the shared interests and participatory action required for its cumulative protection, Mutual Privacy is then classified as an aggregate shared participatory public good which is protected through the group right to Mutual Privacy.

## Introduction

Privacy is in need of conceptual engineering. The collective harms suffered because of contemporary privacy challenges such as group profiling and mass surveillance which cannot be adequately addressed by the traditional conception of privacy as an individual’s right to be let alone (Warren & Brandeis, 1890). Increasingly, when it comes to data processing, the individual is incidental, and the focus of Big Data Analytics is on the group (Taylor et al., 2017a). The conventional formulation of the individual right to privacy is focused on protecting personal data and does not safeguard individual autonomy from exercise of influence and manipulation at group level (Puri, 2021). In order to overcome these conceptual inadequacies, recent works have sought to expand the scope of privacy by examining its group aspects. In the concluding remarks of their seminal work on group privacy,^1^ while discussing the problem of protecting groups created by big data and highlighting the agenda for future research, Taylor et al. point out,

From a pragmatic point of view, it seems undesirable and impossible to grant groups rights if the groups, the criteria for grouping people and their membership could change in a split second. Moreover, if a person is a member of more than a thousand groups at a given moment in time, of which he or she is barely aware. Consequently, it would be an almost Sisyphean task to grant all such groups a right to protect their interests, and therefore even if this is important, **another way must be found. (Emphasis added)**. (Taylor et al., 2017b)

The search for this *another way* is reflected in the subsequent scholarship which has formulated the various conceptions of group privacy (Loi & Christen, 2020; Puri, 2021; Segate, 2022), highlighted privacy dependencies (Barocas & Levy, 2020), articulated privacy as a collective concern (Véliz, 2020) and demonstrated the collective harms suffered on account of privacy violations (Tisné, 2020). While the social value of privacy has been long recognized in privacy scholarship (Regan, 1995), the attempts to secure privacy as a public good have also picked up pace in the recent past (Fairfield & Engel, 2015; Regan, 2016, 2015; Sætra, 2020). This article contributes to this growing field by bringing these related strands of scholarship together through formulation of a group right to Mutual Privacy.^2^ Mutual Privacy is defined as an individual’s interest in securing another person’s privacy to safeguard their shared interests and protect against common vulnerabilities. The article begins by highlighting our various mutually shared interests-genetic, social, and democratic, as well as our common vulnerabilities against algorithmic grouping that give rise to a collective interest in Mutual Privacy. On the basis of the shared interests and participatory action required for its cumulative protection, Mutual Privacy is then classified as an aggregate shared participatory public good which is protected through the group right to Mutual Privacy.

## Mutual Privacy

My search for the *another way* highlighted by Taylor et al. begins with the question: Why should one person have an interest in securing another person’s privacy? The answer lies in our shared common existence, which translates into shared vulnerabilities that can be exploited for data analytics purposes. These vulnerabilities may be the result of shared biological attributes, social relationships, shared information, inferences drawn on the basis of similar lifestyles, democratic interests or algorithmic causal interlinkages drawn on the basis of myriad data points. Regardless of the source of shared vulnerability, an overt focus on individual data protection or individual right to privacy leaves us open to collective privacy harms. For instance, collaborative filtering-based algorithms can be used to recommend movies on the basis of similar movies watched by others (Kearns & Roth, 2020) and also for recommending political content that give rise to filter bubbles (Liu et al., 2021). When one person’s data can be used to undermine another person’s autonomy,^3^ then our privacy becomes “not only interdependent in nature, but also existentially, cumulatively interlinked” (Puri, 2021). An individual’s networked existence gives rise to myriad common interests and innumerable common vulnerabilities. When the myriad common interests and innumerable common vulnerabilities of various individuals are considered together, it results in a groundswell for collective interest in Mutual Privacy that supervenes on individual interests. This bottom-up approach addresses the causal interlinkages and mass inferences that may be drawn at a group level which are beyond the purview of privacy rights aimed solely at protecting individual interests. The complexity and immeasurability of the common interests (Mantelero, 2017; O’Hara & Robertson, 2017) and shared vulnerabilities mean that the collective interest in Mutual Privacy, while being dependent on individual interests is not reducible to individual interests. This approach is in conformity with contemporary scholarship on group privacy which aims to address the risk posed by big data analytics to grouped people at an aggregate level (Taylor et al., 2017a) by developing an account of group privacy that is not reducible to individual interests (Floridi, 2014, 2017; Loi & Christen, 2020). Mutual Privacy provides a plausible solution to the problem highlighted by Taylor et al. by clarifying the relationship between individual and collective interests in privacy.

Other scholars have explored the idea of collective interests leading to a group right to privacy in an algorithmic setting as well (Loi & Christen, 2020; Mantelero, 2017). Mantelero’s conception of collective privacy is aimed at “non-aggregative collective interests” and acts “as the right to limit the potential harms to the group itself that can derive from invasive and discriminatory data processing” (Mantelero, 2017). Loi and Christen examine two conceptions of group privacy, one that is aimed at “groups which have a history of interactions or non-trivially shared goal” and the second in form of inferential privacy aimed at groups defined by a feature. While they consider a group right to privacy plausible in the first sense for “sharing experiences, knowledge, or emotions with insiders, creating a barrier against the gaze and judgment of outsiders”, for the second type of groups they conclude that even if a limited right to privacy is available, it cannot be formulated as a group right in the strong sense because “the allegedly special threat against the inferential privacy of groups…can be reduced to a more familiar problem about harmful uses of generalizable knowledge” (Loi & Christen, 2020). My articulation of Mutual Privacy differs from these important contributions in two ways. Firstly, unlike Mantelero who, while drawing on Newman’s (2004) classification between shared and collective interests, argues for distinction between aggregative interests which are based on interests shared by the entire group without conflict and non-aggregative interests where the individuals might have different opinion, I argue for collective interests based on our common existence and shared vulnerabilities because of which similarity of opinion is not a determining factor. The manifestations of these collective interests may vary but it does not diminish the commonality of our existence and shared vulnerabilities. Secondly, unlike Loi and Christen, who in case of inferential privacy, argue for a limited right to privacy which is not a group right in the strong sense, I argue for a group right to Mutual Privacy which is based on collective interest against algorithmic grouping. This distinction arises out of the moral grounding of Mutual Privacy that emphasizes the larger role of privacy in the protection of interests that are fundamental to our shared common existence. In the ensuing paragraphs, I identify and discuss some paradigm examples of our shared common existence in form of genetic, social, and democratic interests as well as our common vulnerabilities to algorithmic grouping that give rise to the need for Mutual Privacy. These are an illustrative and not exhaustive account of our shared interests and common vulnerabilities that give rise to a collective interest in Mutual Privacy. In view of Mutual Privacy’s emphasis on shared interests and common vulnerabilities that form the bedrock of our existence, its formulation extends to both data protection as well as the larger sphere of autonomy driven account of information privacy.

### Genetic

The collective interest in Mutual Privacy is perhaps most visible in genetic privacy. Lunshof et al. define genetic privacy as “an individual’s right— one that is perhaps extended to families and communities — to protection from nonvoluntary disclosure of genetic information” (Lunshof et al., 2008). The disclosure of genomic data does not impact just the individual but also families and ethnic groups that share genetic similarities (Barocas & Levy, 2020; Gostin, 1995). While it is beyond the purview of the present article to address the various issues pertaining to conflict between individual and family choices regarding use of genetic data, the collective interest in Mutual Privacy is undeniable (Laurie, 2002). Genetic groups are currently not recognized as data subjects for the purposes of data protection (Hallinand, 2021). As a step towards this, from a moral perspective, the considerations of genetic privacy require expansion of horizon from individual interest to collective interest in Mutual Privacy with a focus on participatory and collaborative effort. The collective interest in Mutual Privacy arising out of genetic attributes is based on shared biological attributes, but there are other shared attributes, such as social interests, which give rise to assertions of collective interest in Mutual Privacy in different contexts.

### Social

In privacy scholarship, the social value of privacy has been recognized primarily in three forms—as a common value shared by individuals, as a public value that is important for the functioning of democratic systems and as a common value that is necessary in order to ensure everyone enjoys a minimum modicum of privacy (Hughes, 2015; Regan, 1995). The social value of privacy rests on the role that it plays in an individual’s functioning in society as well as the formation of an individual’s social identity (Regan, 1995; Puri, 2021). The size and type of social group dictates the extent of inferences that can be drawn about the group from the disclosure of information about one member of the group. For instance, in close drawn family settings, location details of a parent may disclose information relating to the child as well. In larger social groups such as race and gender, Big Data Analytics deployed by AI systems may result in proxy discrimination (Schwarcz & Prince, 2020). The shared social interest in Mutual Privacy also arises out of joint custodianship of shared information that requires balancing privacy with disclosure and is vital to managing our relationships (Petronio, 2002). As per Petronio, “[I]nitial disclosures set into motion a need for boundary coordination because there is an expected guardianship of the information often assumed by both the discloser and the recipient” (Petronio, 2002; Regan, 2016). The authorized sharing of information gives rise to co-ownership of information (Petronio & Durham, 2008), which is at the heart of the need to protect Mutual Privacy. Regan states,

On a number of online platforms, in particular social networking sites, one’s own information privacy is dependent upon one’s friends, friends of friends, professional colleagues, fellow members of political and interest groups, those who may have access to one’s information and, perhaps more critically, those whose actions may affect the privacy of others in that group. (Regan, 2019)

This gives rise to a mutual interest in protecting the said information. No matter how discrete an individual is regarding her privacy, technological advances and the voluminous data that is made available by others can be used to make deeply personal inferences about the individual (Tufekci, 2014, 2019). This “networked privacy” requires shared social norms over information sharing (Tufekci, 2019; Marwick & boyd, 2014). Barocas and Levy have highlighted the various forms of privacy dependencies that may arise on the basis of ties, similarities and differences (Barocas & Levy, 2020). They state, “[I]f people are made aware of how their disclosures may implicate close social ties, they may refrain from making such disclosures” (Barocas & Levy, 2020). This shared vulnerability on account of privacy disclosures forms another basis of Mutual Privacy. Recognition of collective interest in privacy leading to the formulation of a group right to Mutual Privacy would be a step towards reclaiming the social value of privacy (Steeves, 2009).

### Democratic

The democratic value of privacy is well recognized under the privacy scholarship (Lever, 2015a, b). Privacy provides the necessary space for the development of dissent and activism, which are necessary for sustaining democracy. All stakeholders of democracy, especially the citizenry, have a collective interest in protecting this democratic value of privacy. One threat to democratic value of privacy arises out of violations of group privacy aimed at political manipulation as witnessed during the Cambridge Analytica scandal (Puri, 2021). Another threat to collective democratic interest emerges from spyware. In July 2021, a global alliance of media outlets released a series of reports raising concerns of surveillance of politicians, human rights activists, journalists and lawyers across the world through a spyware called Pegasus (Kirchgaessner et al., 2021). The revelations raise privacy concerns that go beyond individual violations. These privacy violations were not aimed at select persons in their individual capacities but were a result of calculated surveillance mounted on the basis of their affiliations to groups that form the vanguard of democracy—opposition politicians, activists, journalists etc. In case of privacy violations, the distinction between individual and group interests often gets blurred (Puri, 2021). The individual may be targeted as a proxy for a group. Further, the data curated from the individual can be used to target the group and vice-versa (Puri, 2021). In cases of targeted political surveillance such as Pegasus, even if the individual privacy harms are rectified, it would be insufficient to address the collective harm and chilling effect incurred on account of the mass privacy violation. The alleged surveillance in case of Pegasus is not aimed at just the targeted politician or the journalist, but its revelation has a silencing impact across democratic silos. In such a scenario of targeted political surveillance, the individual right to privacy is woefully inadequate to protect the collective interest in privacy. In the aftermath of another spyware Hermit, Google’s threat analysis group noted,

While use of surveillance technologies may be legal under national or international laws, they are often found to be used by governments for purposes antithetical to democratic values: targeting dissidents, journalists, human rights workers and opposition party politicians. (Sevens & Lecigne, 2022)

When it comes to Big Data Analytics and mass surveillance, privacy is not the only value under threat. Individual liberty and human dignity are increasingly becoming subject to algorithmic determination. The impact of the loss of others’ privacy on public life has been summarized by Rule as,

We should realize that we often stand to gain or lose from widely experienced gains or losses to privacy, regardless of what happens to information about ourselves individually. If nearly everyone around me feels and acts as though all conversations were being overheard, then something crucial is lost from public life-even if I am convinced that my own conversations are secure. (Rule, 2015)

The individual right to privacy cannot address these collective harms to the democratic value of privacy, which leaves the citizens vulnerable to political manipulation and chilling effect.

### Algorithmic Vulnerabilities

In addition to the shared interests highlighted above, individuals are also vulnerable against algorithmic grouping on the basis of demographic attributes and behavioural information for advertisement targeting purposes (Ali et al., 2019). These groupings are rarely targeted at an individual and the data of one individual paves way for targeting other similarly placed individuals through predictive analytics (Mühlhoff, 2021). As Floridi eloquently puts it,

There are very few Moby-Dicks. Most of us are sardines. The individual sardine may believe that the encircling net is trying to catch it. It is not. It is trying to catch the whole shoal. It is therefore the shoal that needs to be protected, if the sardine is to be saved. (Floridi, 2014)

But what is the harm suffered on account of this encircling net, why is the individual right to privacy insufficient to tackle that harm and how can formulation of Mutual Privacy help? These questions can be addressed with the aid of a few examples. Beginning on an economic note, targeted delivery of advertisement can have an exclusionary economic effect with people being discriminated in housing and job opportunities (Ali et al., 2019). Wachter cautions, “if users are segregated into groups and offered or excluded different products, services, or prices on the basis of affinity, it could raise discrimination issues” (Wachter, 2020). From a financial perspective, people’s spending habits reveal information not just about them but also others. Garratt and Oordt state,

[F]irms use information extracted during payments of one consumer without privacy-enhancing techniques to cluster potential future customers into groups with different reservation prices. Hence, failure on the part of one individual to preserve his or her information imposes a negative externality on others. (Garratt & Oordt, 2019)

Since individual failures to maintain financial privacy can lead to “socially suboptimal outcomes”, we must treat privacy as a public good (Garratt & Oordt, 2019). If choices such as spending habits that have been traditionally deemed as personal have a collective privacy implication, then the protection of this shared privacy interest requires a sustained reciprocal effort, which cannot be enabled within the framework of an individual right to privacy.

Our common vulnerabilities against algorithmic grouping are not limited to merely economic and financial parameters but extend to sensitive and personal issues as well. In their paper on the detection of sexual orientation by deep neural networks on the basis of facial images, Wang and Kosinski warn that governments and companies are deploying face-based classifiers aimed at detecting intimate traits (Wang & Kosinski, 2018). The following passage from Wachter and Mittelstadt’s paper on right to reasonable inferences further highlights the need for Mutual Privacy in the age of Big Data Analytics,

Numerous applications of Big Data analytics to draw potentially troubling inferences about individuals and groups have emerged in recent years. Major internet platforms are behind many of the highest profile examples: Facebook may be able to infer sexual orientation—via online behaviour or based on friends—and other protected attributes (e.g., race), political opinions and sadness and anxiety – all of these inferences are used for targeted advertising. (Wachter & Mittelstadt, 2019)

In addition to being violations of individual right to privacy and affront to human dignity, these examples help illustrate the point that an individual’s data does not just belong to her (Véliz, 2020). The individual right to privacy does not capture all the interests at stake where one person’s image serves as the basis for the detection of the sexual orientation of another or where similar inferences can be drawn on the basis of social relations such as friendship. Hence, the need for the recognition of a collective interest in Mutual Privacy which is based on shared interests and common vulnerabilities.

This mosaic effect which is based on “compilation of disparate, often publicly accessible, datasets to create new and potentially sensitive insights” has particularly severe consequences for data concerning vulnerable groups like children (Young, 2020). Privacy scholars have sought to fight back against these intrusive data processing practices by stating, “algorithmically grouped individuals have a collective interest in how information describing the group is generated and used” (Mittelstadt, 2017). However, the problem of protecting the privacy of these algorithmic groups (Kammourieh et al., 2017) is both epistemic and ontological. The epistemic component arises out of the members’ lack of awareness of the existence of these groups (Kammourieh et al., 2017; Mantelero, 2017). The ontological uncertainty arises out of the impermanent nature of the algorithmic groups as well as the sheer number of algorithmic groups that can be created in order to profile individuals (Kammourieh et al., 2017; Pagallo, 2017). For instance, consider the following example by Borgesius,

A person who frequently visits websites about cars and soccer might be profiled as a male sports enthusiast. If that same person books a flight to Amsterdam on a website, advertising for tickets for a game of the local football club, Ajax, may be shown. (Borgesius, 2015)

It is unlikely that the people profiled under the group category ‘male sports enthusiast’ would be aware of their participation in such a group and the consequences thereof. Arguendo, even assuming that the person is aware of the existence of such a group, the present privacy infrastructure does not contain adequate legal and conceptual safeguards to either protest or protect the person’s inclusion in such a group. The group profiling further does not stop but only begins at these broad conceptual levels and increases in granularity. Users are further clustered, and more nuanced algorithmic groups are created on the basis of their stated preferences such as likes (van Dam & Van De Velden, 2015) as well as inferences drawn on the basis of data gathered from other similar users. These groups are not stable but hyperdynamic entities whose membership keeps on changing depending on the frame of reference deployed by Big Data Analytics. In view of the epistemic and ontological constraints associated with algorithmic groups, these groups themselves may not be able to stake a claim against inferential privacy (Loi & Christen, 2020). Hence, we must treat the creation of these profiling groups as an exploitation of our common vulnerabilities and assert our collective interest in Mutual Privacy.

Further evidence of the need for Mutual Privacy emerges from advances in advertising technology that outline shared privacy interests in the context of algorithmic grouping. Google’s now-abandoned Federated Learning of Cohorts (FLoC) initiative was proposed as “a new way for businesses to reach people with relevant content and ads by clustering large groups of people with similar interests” (Bindra, 2021). As Boiten put it, “[R]ather than tracking and targeting you on an individual basis, Google’s alternative groups you instead into a crowd of people with similar generalised interests” (Boiten, 2021). FLoC’s tryst with Mutual Privacy becomes clearer with the aid of this extract from FLoC proposal on GitHub,

We plan to explore ways in which a browser can group together people with similar browsing habits, so that ad tech companies can observe the habits of large groups instead of the activity of individuals. Ad targeting could then be partly based on what group the person falls into. (FLoC, 2019)

FLoC came under stringent criticism from the Electronic Frontier Foundation (EFF) which had raised concerns that FLoC may “exacerbate many of the worst non-privacy problems with behavioural ads, including discrimination and predatory targeting” (Cyphers, 2021). EFF further flagged concerns relating to unsupervised algorithm being at the helm of the creation of clusters and the high likelihood of grouping people on the basis of sensitive characteristics such as gender, age and mental health (Cyphers, 2021). FLoC was subsequently abandoned (Nguyen, 2022). Researchers at MIT Media Lab in their assessment of FLoC after its cancellation have stated that “FLoC would have enabled individualized cross-site user tracking by providing a unique identifier for users available across sites, similar to the third-party cookies FLoC was meant to be an improvement over” (Berke & Calacci, 2022). Google’s subsequent privacy initiative Topics API has also been criticized for being a rebranded version of FLoC without addressing the key privacy issues (Snyder, 2022) and for continuation of inappropriate surveillance on the web (Lomas, 2023, W3C TAG, 2022).

While the aforementioned privacy solutions in advertising technology may have some impact in terms of invasive personalized surveillance at the individual level, they do little to assuage concerns of violation of individual autonomy on account of manipulation suffered because of excessive data collection. This is because many of the existing technical solutions are based on a flawed understanding of privacy as Personally Identifiable Information. Whether for data collection purposes, the epistemic predicate of attending a music concert, suffering from an illness and belonging to a political ideology is targeted directly at the individual or at the group to which the individual belongs; the result from Big Data Analytics perspective would remain the same. It would allow the drawing of causal inferences for behavioural targeting purposes, which can then be used to undermine individual’s autonomy. When it comes to Big Data Analytics in the realm of advertising technology, an individual is merely an aggregation of groups to be panoptically sorted (Gandy, 1993). An individual’s autonomy can be violated even without infringing her anonymity (Barocas & Nissenbaum, 2014, Puri, 2021). If an individual can still be discriminated against, her worldview still be distorted, and her control over her social identity formation still be compromised; then, it matters little whether the privacy violation is taking place on lines of player-to-player marking or zone defence (Puri, 2021).^4^ As long as privacy will continue to be defined through the narrow lens of individual identification, any corresponding technological or regulatory measures would be inadequate to protect individual autonomy and identity. Hence, there is a pressing need for assertion of a collective interest in Mutual Privacy. The urgency of this need can be further understood with the aid of the limitations of the individual rights approach in face of the collective privacy harms.

## Limitations of the Individual Rights Approach in face of the Collective Privacy Harms

Individual autonomy is the bedrock of the liberal tradition. The exercise of this autonomy is done through consent. From a moral perspective, an individual can be said to be capable of exercising her rights if the negative consequences thereof are limited to her. However, when an individual’s privacy choices start impacting other people’s autonomy on account of negative externalities and information leakages, the limitations of the individual consent model become evident (Sætra, 2020). In view of the limitations of the individual consent model, “data protection rights need to be extended to allow for data rights to be managed collectively” (Ruhaak, 2020). Tufekci states,

Data privacy is not like a consumer good, where you click “I accept” and all is well. Data privacy is more like air quality or safe drinking water, a public good that cannot be effectively regulated by trusting in the wisdom of millions of individual choices. A more collective response is needed. (Tufekci, 2018)

The realization of the need for collective response is also driven by collective harms that are incurred on account of privacy violations. The sorting of people into groups by Big Data Analytics leads to collective harms in form of discrimination and inequality (Aaronson, 2021; Obar & Mcphail, 2018). Mulligan et al. state “Protecting the collective, public, and social value of privacy requires us to refocus on addressing collective harms, as well as individual ones, that flow from surveillance practices” (Mulligan et al., 2020). The collective concern arising out of privacy violations has become further imperative in view of the “turbo-digitalization” caused by the COVID-19 pandemic (Mühlhoff, 2020). Tisné states, “[T]he collective nature of big data means people are more impacted by other people’s data than by data about them. Like climate change, the threat is societal and personal” (Tisné, 2020). The collective nature of privacy harms means that the individual consent model is inadequate to address the challenge posed by Big Data Analytics and requires recognition of Mutual Privacy as a public good.

## Mutual Privacy as a Public Good

The realization of collective aspects of privacy has led to a growing recognition of privacy as a public good. The articulation of privacy as a public good is aimed at complementing individual privacy with a collective formulation (Rusinova & Pereverzeva, 2020). While staking a claim that perhaps we have been understanding privacy the wrong way around, Kieron O’Hara states, “…privacy isn’t a private benefit like health or champagne, but a public good like clean air or scientific research. If so, giving away our privacy might be similar to polluting the atmosphere or refusing to publish our results” (O’Hara, 2013). Fairfield and Engel highlight that an individual may be vulnerable on account of the privacy disclosure of others (Fairfield & Engel, 2015). They further argue that privacy is a public good and its protection requires group coordination. In view of the public good nature of privacy, the conceptual framework of privacy must be aimed at empowering groups (Fairfield & Engel, 2015). However, like all public goods, the collective interest in privacy can suffer from the free-rider problem. This would mean that an individual might choose to compromise their own data in order to receive the perceived benefits of accessing a website, while hoping that others would continue to be vigilant with their data choices. Our ability to mitigate the free-rider problem depends on the nature of privacy as a public good and the strategies adopted to protect it.

### The Free-Rider Problem

Public goods are non-excluding and non-rivalrous but suffer from the free-rider problem (Cowen, 2008). Fairfield and Engel attempt to overcome the free-rider problem of privacy as a public good by highlighting the framing effect in terming privacy as a public good as opposed to considering a lack of privacy as a public bad (Fairfield & Engel, 2015).

The articulation of Mutual Privacy helps contribute to the solution of free-rider problem in three ways: firstly, emphasis on common existence can lead to more awareness about individual privacy choices. The focus on shared interests in privacy in public discourse would result in enhanced co-operation. If an individual were to be made cognizant of the privacy harms suffered by others on account of the information shared by them, not only would they be more circumspect in sharing their information but from a group dynamic perspective, in view of the repeated interaction between members in a group, there would be a greater incentive to co-operate (Fairfield & Engel, 2015). Secondly, the articulation of Mutual Privacy as an aggregate shared participatory good, in the next section, highlights the extent of co-operation required for protection of Mutual Privacy; and lastly, the articulation of group right to Mutual Privacy can serve as a policy tool and co-ordination mechanism for overcoming the free-rider problem.

### Mutual Privacy as an Aggregate Public Good

While classifying Mutual Privacy as a public good and devising strategy to protect the collective interest in privacy, it would be helpful to remember that protection of each individual privacy has a net positive impact across all other individual privacies. This realization helps us understand Sætra’s assertion that “[P]rivacy is a good of the aggregate type, as no single actor can solve it alone but it does not require all to take part in its provision” (Sætra, 2020). The same point can only be understood through the perspective of aggregate harm. In view of the Internet’s importance as a public service infrastructure, the privacy harm is not only restricted to the individual, but the aggregate loss extends to the public infrastructure as well (Lao & Baker, 2021). The aggregate nature of Mutual Privacy helps explain the need for collective action to protect privacy; however, it does not fully explain the shared interests in privacy. The shared interest aspects of Mutual Privacy become clearer with its analysis as a shared and participatory public good.

### Mutual Privacy as a Shared and Participatory Public Good

As per Raz, “[A] good is a public good in a certain society if and only if the distribution of its benefits in that society is not subject to voluntary control by anyone other than each potential beneficiary controlling his share of the benefits” (Raz, 1986). A public good is also non-excluding (Raz, 1986). But from a privacy perspective, neither the non-excluding nor the non-rivalrous aspect of a public good helps explain the mutually shared interests that arise when the data of one individual can be used to influence the choices of others (Barocas & Levy, 2020; Puri, 2021). Hence, we need to look at sub-classes of public goods that can help explain this aspect of privacy.

The mutually shared interest in privacy arises out of shared attributes, sharing of information or the joint participation of individuals in the public sphere, which can help us understand privacy as a shared participatory good. As per Raz, “[S]hared goods are goods whose benefit for people depends on people enjoying the good together and thereby contributing to each other’s good” (Raz, 1995). Slater cites team sports as an example of the cooperation required to exercise valuable autonomy in shared goods (Slater, 2017). This is interesting because one of the earliest examples of group privacy, cited by Bloustein, is the strategy jointly developed in a football huddle in which all team members have a collective interest to protect (Bloustein, 1977). Green cites friendship as an example of a shared good “which can be enjoyed only in a form of association which itself partly constitutes the good shared” (Green, 1988). As per Green, shared goods “are like public goods except that their public aspect is not merely a contingent feature of their production but partly constitutes what is valuable about them” (Green, 1991). This formulation of shared goods resonates strongly with the social value of privacy, which has been highlighted by Regan and other scholars (Regan, 1995; Steeves, 2009). Privacy is not just an individual right but also a social value, which in the age of Big Data Analytics, can be meaningfully protected only through a collective effort (Puri, 2021).

Participatory goods as defined by Reaume, “involve activities that not only require many in order to produce the good but are valuable only because of the joint involvement of many” (Réaume, 1988). From Mutual Privacy’s perspective, there are two ways to examine this participation. First, Mutual Privacy facilitates public participation. When individuals are secure about the privacy of their communication, they can express themselves freely. Second, the joint custodianship of shared information is itself a valuable public good that is arising out of participation. These attributes lead us towards the classification of Mutual Privacy as a participatory public good. This formulation helps address the free-rider problem identified by Fairfield and Engels (Fairfield & Engel, 2015). As Reaume states, participatory goods avoid the free-rider problem because they unite production and consumption (Réaume, 1988). A participatory good doesn’t depend on any end product but is constantly recreated and re-interpreted (Réaume, 1988). Conceptualization of Mutual Privacy as a participatory public good means that it would be difficult for an individual to continue to be reckless with their own individual privacy choices and continue to enjoy the benefit of others’ collaborative efforts to protect the collective interest in privacy. One’s reckless privacy choices, while causing proximate harm to the collective interest in privacy, cause ultimate harm to one’s individual interests (Raz, 1995).

Mutual Privacy is an act of participatory creation and joint custodianship. While scholars agree on the conceptual formation of participatory goods, they disagree over who has a right to participatory goods- individuals, collectives or groups (Goemans, 2018; Morauta, 2002; Réaume, 1988). For Mutual Privacy purposes, it suffices that the participatory nature of privacy supplements our conventional understanding of individual privacy. Its enforcement may be done in an individual capacity (Morauta, 2002) or as a representative group capacity (Goemans, 2018).

There is a degree of overlap in classification of Mutual Privacy as a shared public good and as a participatory public good, as the two classifications are aimed at highlighting two related aspects of collective interest in privacy. The classification of Mutual Privacy as a shared public good is aimed at highlighting the shared interests that give rise to collective interest in Mutual Privacy. The classification of Mutual Privacy as a participatory public good is aimed at highlighting the reciprocal effort required to protect the collective interest in Mutual Privacy.

In light of the aforesaid discussion surrounding aggregate, participatory and shared goods, we can conceptualize *Mutual Privacy as an aggregate shared participatory public good*. Mutual Privacy is created through a participatory process and is strengthened through each act of individual privacy protection. The collective interest in Mutual Privacy can only be enjoyed together and by contributing to the protection of other people’s privacy.

## The Group Right to Mutual Privacy

In the previous sections, I have identified the collective interests that form the basis of Mutual Privacy as an aggregate shared participatory public good. This part is devoted to converting those collective interests into a group right. In order to do so, it would be important to examine the nature of collective group rights for the requirement of an identifiable membership of a group. Group rights are generally divided into corporate rights—the rights which are held by the group themselves and collective rights—the rights which are an assertion of collective interest (Jones, 2016). Collective group rights pertain to a collection of individuals “that is bound together in a way that enables them to hold their right collectively” (Jones, 2016). If corporate and collective groups are the traditional subjects of group rights, then public goods are the conventional objects of group rights (Jones, 2016). When it comes to the public good of Mutual Privacy, the need for a collective interest-based response in form of a group right can be understood with the aid of Fairfield and Engel’s assertion “[G]roups must be given tools to create the public good of privacy and resist the public bad of readily available intrusive information” (Fairfield & Engel, 2015). The relevant tool required for protecting Mutual Privacy would be a group right which is based on collective interest. As per Floridi, “Privacy as a group right is a right held by a group as a group rather than by its members severally. It is the group, not its members, that is correctly identified as the right-holder” (Floridi, 2014). But does this group need to be a stable ontological entity or would an assertion of the collective interest suffice even in the absence of an identifiable membership?

To address this question, it would be helpful to begin our inquiry at the level of individual interests and work towards collective interests. As regards an interest-based exposition of individual rights, Raz states, “‘X has a right’ if and only if X can have rights, and, other things being equal, an aspect of X’s well-being (his interest) is a sufficient reason for holding some other person(s) to be under a duty” (Raz, 1986). However, for Raz, “the interests which justify the right and which give it its shape and content are the interests of the public at large alongside the interests of the rightholder” (Raz, 1995). Further, when it comes to collective right to a public good, Raz imposes the following three conditions,

First, it exists because an aspect of the interest of human being justifies holding some person(s) to be subject to a duty. Second, the interests in question are the interests of individuals as members of a group in a public good and the right is a right to that public good because it serves their interest as members of that group. Thirdly, the interests of no single member of that group in that public good is sufficient by itself to justify holding another person to be subject to a duty. (Raz, 1986)

Jones disagrees with this formulation, “[B]ut it is not clear why we should hold that a set of individuals can have a collective right only if they are antecedently identifiable as members of a group” (Jones, 2016). Jones has sought to modify Raz’s formulation by stating,

Any set of individuals who possess a joint interest in a good can have group rights relating to that good provided that their joint interest is sufficiently significant to create duties for others. What unites and identifies a set of individuals as a group for right-holding purposes is simply their possessing a shared interest of sufficient moment. (Jones, 1999)

From a Mutual Privacy perspective, the emphasis on membership in a group in Raz’s initial formulation while accounting for recognizable social groups runs the risk of excluding other shared interests from the collective assertion of the public good of Mutual Privacy. This also goes against Regan’s assertion of there being a collective interest in privacy (Regan, 2002). Further as highlighted by Taylor et al., in case of algorithmic groups, since the formation of the group itself is an act of violation of privacy and the individual is part of innumerable such groups, the assertion of Mutual Privacy on the basis of these memberships would be impractical (Taylor et al., 2017b). Hence, while articulating the group right to Mutual Privacy, an overt emphasis on group membership is neither desirable nor feasible. The group right to Mutual Privacy is not based on membership in a group but on a collective interest in Mutual Privacy and can be defined as:

A group of individuals have a collective interest in safeguarding their shared interests and protecting against common vulnerabilities, which other things being equal is a sufficient reason for holding some other person(s) to be under a duty to refrain from violating their Mutual Privacy.

This formulation of the group right to Mutual Privacy on the basis of shared interests and common vulnerabilities helps address the ontological and epistemological challenge that hyperdynamic algorithmic groups face in securing their privacy. Since the collective interest in Mutual Privacy is neither conditioned upon membership in “a group” nor is it conditioned upon existence of “the group”, it does not suffer from the same constraints as algorithmic groups. I share the concern expressed by Loi and Christen that an inferential right to privacy in group setting cannot be absolute (Loi & Christen, 2020). Hence, the formulation of group right to Mutual Privacy has a balancing provision in form of “other things being equal”. This can help us balance, for instance, privacy interests with interests in scientific research while thwarting nefarious attempts at group profiling for commercial exploitation purposes.

### The Relationship Between the Group Right to Mutual Privacy and the Individual Right to Privacy

The relation between individual and group interests can be understood from multiple perspectives. When it comes to justification for group interests Raz states,

First, groups as well as individuals possess rights. Group rights, the rights of nations, families, and the like, are based on the interests of these groups. Naturally, there is no intrinsic value in protecting the interests of groups. Their interests merit protection only to the extent that they serve individual interests. Whatever the ultimate justification of group rights, they are the rights of groups and not of individuals. Nor do they derive their justification from individual rights; rather, their proximate justification is in the interest of the group, and their ultimate justification lies in the service to individual interests of advancing the interest of the group. (Raz, 1995)

Understood this way, the proximate justification of the group right to Mutual Privacy lies in the collective interest in Mutual Privacy, and its ultimate justification lies in the service to individual interests of advancing the collective interest in Mutual Privacy (Raz, 1995). Another way of understanding the relationship between individual and collective interest has been articulated by Newman; “[C]ertain individual interests that ground duties are meaningful interests and can be fulfilled only on the precondition that certain collective interests are also rights” (Newman, 2004). While Newman’s articulation of collective interest differs from mine, when it comes to privacy, I agree that its meaningful protection requires recognition of both individual as well as collective interest. This is also in line with Snowden’s assertion that “[W]e need to recognize that people have an individual right to privacy but they also have a collective right to privacy” (Rusbridger & MacAskill, 2014). In the conclusion of their work on group privacy, Taylor et al. have proposed it “as an enhancement and safeguard for the individual right to privacy, rather than as a potential substitute for it” (Taylor et al., 2017b). Véliz states “Privacy is both personal and collective. When you expose your privacy, you put us all at risk” (Véliz, 2019a). She further highlights, “Privacy protects us from both individual and collective harms” (Véliz, 2019b). Hence, we need not conceptualize the group right to Mutual Privacy in contradistinction to the individual right to privacy. The group right to Mutual Privacy complements the individual right to privacy.

## Conclusion

We are defined by the values that we care for and the interests we deem worthy of protecting.^5^ In this article, I have highlighted the limitations of the individual model of privacy in light of the contemporary challenges to privacy and developed a response to these challenges on the basis of our collective interests in Mutual Privacy. The conceptual foundations of Mutual Privacy rest on the realization that in a hyper-connected world, privacy harms are not limited to the individual but extend to the collective. In the age of group profiling and mass surveillance, the creation, sustenance and protection of privacy are no longer an individual concern but a collective interest that needs to be protected as an aggregate shared participatory public good through the group right to Mutual Privacy. The formulation of the group right to Mutual Privacy apart from acting as a conceptual safeguard also increases the normative weight of privacy. From a policy perspective, it can help shift the focus of the debate from individual privacy v. commercial interests to collective privacy interests v. commercial interests (Puri, 2021). The moral group right to Mutual Privacy articulated in this article is the first step towards conceptually engineering privacy. Its legal recognition can help expand the focus of the privacy regulatory landscape from the individual to the collective. These are the steps that we need to take towards the *another way* that was sought in the beginning of the article.

## Data Availability

I do not analyse or generate any datasets, because my work proceeds within a theoretical approach.

## Footnotes

1 While addressing multiple aspects of group privacy from the perspective of different disciplines, Taylor et al. adopt an approach that is “functional and iterative rather than stable and unanimous”. Hence, instead of a homogenous account of group privacy various nuances emerge from different perspectives. The guiding principle of their scholarship is a search to move the analysis of group privacy “beyond a collection of individuals with individual interests in privacy” i.e. from “their privacy” to “its privacy” in context of a group (Taylor et al., 2017a).

2 “Mutual or companion privacy means that not only our privacy is interdependent, in fact a significant portion of my privacy resides in yours. The only way of ensuring my privacy is by saving your privacy” (Puri, 2021).

3 In view of our common existence and shared vulnerabilities against Big Data Analytics, the account of autonomy referred to in this article is that of relational autonomy which is defined “in terms of what is at issue for individuals who are situated with others in the real-world context of moral, social, and political exchange. Accepting this means that a plausible account of autonomy must abandon the premise that autonomous agency is conceived in atomistic or individualistic terms” (Oshana, 2020).

4 The sports defence strategy of player to player marking and zone defence are useful analogies to explain the working of individual right to privacy and the group right to Mutual Privacy vis-à-vis Big Data Analytics. Player to player marking in sports refers to individual defence players tracking individual opponent strikers in order to prevent them from scoring, whereas in zone defence, each player is asked to focus on defending a particular area of the field (Masterclass Staff, 2020). Since it is clear that the individualistic approach to privacy protection is not working, we need to shift our focus to “zone defence” and adopt a collectivist approach in form of Mutual Privacy.

5 As St. Augustine poetically wrote, “[A] people is the association of a multitude of rational beings united by a common agreement on the objects of their love.” (Lapointe, 2016).
